# Mitochondrial DNA methylation is a predictor of immunotherapy response and prognosis in breast cancer: scRNA-seq and bulk-seq data insights

**DOI:** 10.3389/fimmu.2023.1219652

**Published:** 2023-06-29

**Authors:** Yixuan Ma, Juan Du, Meini Chen, Ning Gao, Sijia Wang, Zhikuan Mi, Xiaoli Wei, Jumei Zhao

**Affiliations:** Shaanbei Key Laboratory for Cancer Prevention of Yan’an, Medical College, Yan’an University, Yan’an, China

**Keywords:** breast cancer, mitochondrial DNA methylation (MTDM), NCAPD3, immunotherapy, prognostic model

## Abstract

**Background:**

Alterations in Mitochondrial DNA methylation (MTDM) exist in many tumors, but their role in breast cancer (BC) development remains unclear.

**Methods:**

We analyzed BC patient data by combining scRNA-seq and bulk sequencing. Weighted co-expression network analysis (WGCNA) of TCGA data identified mitochondrial DNA methylation (MTDM)-associated genes in BC. COX regression and LASSO regression were used to build prognostic models. The biological function of MTDM was assessed using various methods, such as signaling pathway enrichment analysis, copynumber karyotyping analysis, and quantitative analysis of the cell proliferation rate. We also evaluated MTDM-mediated alterations in the immune microenvironment using immune microenvironment, microsatellite instability, mutation, unsupervised clustering, malignant cell subtype differentiation, immune cell subtype differentiation, and cell-communication signature analyses. Finally, we performed cellular experiments to validate the role of the MTDM-associated prognostic gene *NCAPD3* in BC.

**Results:**

In this study, MTDM-associated prognostic models divided BC patients into high/low MTDM groups in TCGA/GEO datasets. The difference in survival time between the two groups was statistically significant (P<0.001). We found that high MTDM status was positively correlated with tumor cell proliferation. We analyzed the immune microenvironment and found that low-MTDM group had higher immune checkpoint gene expression/immune cell infiltration, which could lead to potential benefits from immunotherapy. In contrast, the high MTDM group had higher proliferation rates and levels of CD8+T cell exhaustion, which may be related to the secretion of GDF15 by malignant breast epithelial cells with a high MTDM status. Cellular experiments validated the role of the MTDM-associated prognostic gene NCAPD3 (the gene most positively correlated with epithelial malignant cell proliferation in the model) in BC. Knockdown of NCAPD3 significantly reduced the activity and proliferation of MDA-MB-231 and BCAP-37 cells, and significantly reduced their migration ability of BCAP-37 cell line.

**Conclusion:**

This study presented a holistic evaluation of the multifaceted roles of MTDM in BC. The analysis of MTDM levels not only enables the prediction of response to immunotherapy but also serves as an accurate prognostic indicator for patients with BC. These insightful discoveries provide novel perspectives on tumor immunity and have the potentially to revolutionize the diagnosis and treatment of BC.

## 1 Introduction

Breast cancer (BC) is one of the most prevalent cancers that threatening women’s lives and health. According to Global Cancer Statistics 2020 (1), BC has surpassed lung cancer as the most common type of tumors in women. According to statistical data, nearly 2.3 million new BC cases were reported in 2020, accounting for 11.7% of all cancer cases. Similarly, a study in 2022 showed that the mortality rate of BC in China will also increase dramatically, making it the most common cancer among Chinese women (2). BC is further classified into luminal (estrogen receptor [ER] positive), human epidermal growth factor receptor 2 (HER2) positive and ER-negative, and basal subtypes (3), and targeted therapies based on molecular typing have been applied clinically, changing the previous “one-size-fits-all” treatment approach (4). Over the past ten years, innovative healing methods, such as immunotherapy, have progressed remarkably. By utilizing the patient’s immune system to detect and regulate tumors, immune checkpoint inhibitors (ICIs) such as PD-1, PD-L1, and CTLA-4 have effectively enhanced the prognosis of different types of cancers (5). However, recent clinical trials have shown that combined therapies are often more effective than single immunotherapy for BC (6), suggesting that more effective immunotherapy markers are needed to enable BC patients to benefit from immunotherapy. Currently, PDL1 may not be an ideal marker to identify patients sensitive to immunotherapy, as PD-L1 expression is dynamic and varies not only between individuals but also over time (7). Targeting mitochondria is a new option for tumor immunotherapy. Recent evidence suggests that using anti-cancer drugs to target the mitochondrial pathway can greatly enhance the ability of cancer cells to be recognized by immune cells, present tumor antigens, and enhance the anti-tumor function of immune cells, leading to the effective killing of cancer cells (8, 9). Thus, exploring the correlation between various biological pathways within mitochondria and the development of tumors, as well as the immune microenvironment, would be worthwhile. This will provide valuable insight into the mechanisms underlying tumorigenesis and facilitate the development of more effective therapies.

Mitochondria are essential organelles that control not only cellular energy metabolism but also the main site of energy metabolism; they are essential organelles for regulating reactions such as calcium homeostasis, apoptosis, and redox reactions, thus maintaining the homeostasis of the internal environment and playing a crucial role in cancer development. Mitochondrial DNA (mtDNA) is a double-stranded loop, divided into heavy (H) and light (L) strands, without histone involvement, and its circular DNA contains three promoter regions, located in the D-loop, transcribed as multiple cis-trans (10), which encode 13 oxidative phosphorylation (OXPHOS) subunits of proteins, as well as two ribosomal RNA genes and 22 tRNAs (11). These 13 proteins encoded by the mitochondria are components of the electron transport chain and are involved in regulating the oxidative respiratory chain and maintaining its functional integrity; alterations in their expression have been associated with cancer development (12–14). In addition, mitochondria play a key role in immune system functioning by regulating the development, activation, proliferation, differentiation, and death of immune cells. For example, mitochondria control immune cell differentiation by regulating metabolism and mtROS production (15, 16).

Recently, mitochondrial epigenetics has gained much attention, and the biological function of Mitochondrial DNA methylation (MTDM) has gradually been explored. Altered levels of mtDNA methylation and hydroxymethylation have been observed in various diseases, including BC, cardiovascular disease, diabetes, and neurodegenerative diseases (17). MTDM lacks CPG islands and methylates non-CPG sites such as CPA, CPC, and CPT (18), which may be associated with the development of various diseases. Initially, the role of MTDM was controversial, but as research progressed, the mitochondrial isoform of *DNMT1* (mtDNMT1) was identified in mitochondria which is homologous to nuclear *DNMT1*, confirming that *mtDNMT1* binds to the D-loop control region of mitochondria and forms a 5-mC that regulates mitochondrial gene expression (19, 20). Subsequently, *DNMT3A* and *DNMT3B* were found to be involved in MTDM (21, 22) which is associated with the active methyl donor SAM. Therefore the expression or absence of *SLC25A26*, the only channel for SAM entry into the mitochondria, also controls the level of MTDM (23). Despite significant attention in recent years, the precise role of MTDM in BC and its impact on the immune microenvironment remain unclear. To address this knowledge gap, our study employed both single-cell and bulk sequencing to investigate the biological function of MTDM in BC, as well as its potential as a prognostic indicator and immunotherapeutic marker. By shedding light on the implications of MTDM, we hope to understand BC’s development and progression better while offering new avenues for treatment and patient care.

## 2 Method

### 2.1 Transcriptome data download and processing

TCGA data of the training cohort was downloaded by using the “TCGAbiolinks” R package, with the TPM data type selected. Considering that more than 99% of BC patients were female, 13 male patients were excluded to maintain data integrity, and survival times ranged from 3 to 120 months for BC patients. Ultimately, 945 tumor samples were included in this study. The BC dataset GSE21653, downloaded from the GEO database (24) was used as the validation cohort. For subsequent analyses, all data were log2 transformed.

### 2.2 Single-cell sequencing data download and processing

The single-cell BC dataset GSE195861 (25) was downloaded from the GEO database, with samples of ductal carcinoma *in situ* (DCIS) and Invasive Ductal Carcinoma (IDC). The Seurat package was used for subsequent processing, and data quality control was performed by selecting cells with ribosomal genes ranging from 0 to 50, with a total number of genes greater than 200, and genes expressed in at least ten cells. The number of highly variable genes was set to 3000, and samples with a cell count greater than 500 were selected after filtering. They were then corrected and integrated by the IntegrateData function, followed by cells being clustered by setting the “DIMS” parameter to 20, reducing the data dimension using the UMAP method, and setting the resolution to 0.2 using the K-NearestNeighbor (KNN) method. Cell markers were then downloaded for annotation using the CellMarker 2.0, website (http://yikedaxue.slwshop.cn/), and each cell’s percentage of MTDM genes was obtained by importing the MTDM-mediated genes through the PercentFeatureSet function.

### 2.3 Acquisition of mitochondrial DNA methylation -related genes

*DNMT1*, *DNMT3A*, *DNMT3B*, *SLC25A26*, *METTL4*, *NRF1*, *PPARGC1A* and *PRKAA1* were identified as MTDM-related genes according to the literatures (20–23, 26, 27)

### 2.4 Weighted co-expression network analysis and single sample gene set enrichment analysis

Co-expression network of all genes in MTDM and BC samples was constructed using the TCGA breast cancer patients’ data cohort and the “WGCNA” R package. Genes in the top 90% were selected by variance screening and outlier sample filtering. The pickSoft Threshold function was used to calculate the optimal threshold of 10 and to set the minimum number of module genes to 80, which was then used to construct the WGCNA network. The R package GSVA (28) obtained scores associated with MTDM for each BC patient using the ssGSEA analysis module. Finally, the correlation between the modules and the MTDM scores in the WGCNA network was calculated.

### 2.5 Copynumber karyotyping analysis within tumors and mitochondrial DNA methylation differential biological pathway analysis

Subclonal structure analysis was performed using the Copykat package (29) to distinguish between normal and malignant cells, and the irGSEA package was used to score the high and low MTDM groups using AUCell, UCell, single-score, and ssGSEA enrichment methods. A heat map of the differentially enriched pathways between the two groups was generated.

### 2.6 Construction of mitochondrial DNA methylation -related prognostic model and external validation of the model

Univariate COX analysis was used to identify MTDM-related genes with prognostic value, which were further screened using least absolute shrinkage and selection operator (LASSO) regression to construct prognostic models. This approach allowed the calculation of MTDM scores for each BC sample by multiplying the coefficients by the expression and accumulating the results. Patients in the TCGA BC cohort were divided into high and low-risk groups based on the median value. Subsequently, the prognostic differences between the two groups were explored, and the accuracy of the model was assessed. The GSE21653 cohort in GEO was selected as the external validation cohort, MTDM scores for each sample were calculated according to the model formula and patients were divided into high-risk and low-risk groups based on the median. Survival analysis was then performed to determine whether the prognosis differed between the high- and low-risk groups in the validation cohort, with ROC curves used to assess the accuracy of the model. Principal component analysis (PCA) was used to explore whether the model could better group high and low MTDM, and a Nomogram was constructed through the rms package to assess the risk of death in patients with BC by combining clinical data with MTDM values. Finally, the accuracy of the nomogram for estimating patient outcomes was assessed using prognostic ROC curves.

### 2.7 Immune infiltration and the use of relevant scores

Immune infiltration analysis using the R package IOBR (30) was performed on BC samples using the CIBERSORT, EPIC, MCP-counter, Quanti-seq, TIMER, xCell, and ESTIMATE methods, with the differences in immune cell infiltration between different MTDM subgroups explored in the form of heat maps that showed the immune cells at different infiltration levels. Additionally, differences in tumor mutation burden (TMB) and microsatellite instability (MSI) between the different MTDM subgroups were explored to investigate the sensitivity of patients in different subgroups to immunotherapy. TMB was analyzed using the maftools package (31), whereas MSI was analyzed using the PreMSIm package analysis (32), with the data normalized from 0 to 1.

### 2.8 Calculate the proliferation score

Proliferation scores were calculated using proliferation-related genes (*BIRC5*, *CCNB1*, *CDC2*, *NUF2*, *CEP55*, *NDC80*, *MKI67*, *PTTG1*, *RRM2*, *TYMS*, and *UBE2C*) *(* 33) and grouped using scRNA-seq and bulk-seq.

### 2.9 Analysis of intercellular interactions

Cell-cell interaction analysis was performed using the R package CellChat (34), which models the probability of intercellular communication by combining gene expression with *a priori* knowledge of the interactions between signaling ligands, receptors, and their cofactors utilizing secretory signaling and cell-cell contact human databases. Circle and bubble plots were used to show the strength of cell-cell communication networks from target cell clusters to other cell clusters.

### 2.10 Cell culture and transfection

MDA-MB-231 and BCAP-37, were provided by the Medical Experiment Center of Yan’an University and cultured in RPMI-1640 (Biological Industries, Kibbutz Beit-Haemek, Israel) or DMEM (Biological Industries, Kibbutz Beit-Haemek, Israel) medium supplied with 10% fetal bovine serum (FBS) (Biological Industries, BI) at 37 °C, 5% CO_2_. For small interference RNAs (siRNAs) transfection, jetPRIME reagent (polyplus-transfection, SA) was used according to the manufacturer’s protocol. siRNAs of NCAPD3 were produced by GenePharma (Shanghai, China). The sequences of NCAP3 siRNAs is si-NCAPD3-1, forward 5′- GCAUUCAGACUCUAAAGAATT -3′, and reverse 5′- UUCUUUAGUCUGAAUGCTT -3′; si-NCAPD3-2, forward 5′-GAGAAGGAGAUAAGGUCAUTT-3′, and reverse 5′-AUGACCUUUAUCUCCUUCUCTT-3′.

### 2.11 Quantitative real-time PCR

Total RNA was extracted using TRIzol reagent (invitrogen, Carlsbad, CA, USA), followed by a reverse transcription using a reverse transcription kit (TaKaRa, RR036A) according to the manufacturer’s instructions. Quantitative real-time PCR was performed using a qRT-PCR reagent (TaKaRa, RR820A) according to the manufacturer’s instructions. *β-ACTIN*, was used as the interference gene. Relative expression changes of genes were calculated using the 2^-ΔΔCt^ method. Primer sequences used are *NCAPD3*, forward 5’- TGGAGCAAGAGTCGAATGGCG -3′ and reverse 5′- GGGGCGGTTTATCAGGCAGTG -3’; *β-ACTIN*, forward 5′- CATGTACGTTGCTATCCAGGC -3′, and reverse 5′- CTCCTTAATGTCACGCACGAT -3’.

### 2.12 Western blot analysis

Whole cell proteins were first extracted on ice using the RIPA lysis buffer with protein inhibitors. And then, the proteins extracted were quantified using a BCA protein assay kit (Beyotime, P0010S), 8% SDS-PAGE gels separated, and transferred to a PVDF membrane. For western bloting analysis, the PVDF membranes were then 5% skim milk blocked, primary antibody blotted, TBST washed, secondary antibody incubated, TBST washed and chemiluminescence detected. Primary antibodies of NCAPD3 antibody (Proteintect, Wuhan, China, 16828-1-AP) and *β*-Tubulin antibody (Proteintect, Wuhan, China, 10094-1-AP), and HRP tagged rabbit secondary antibody (Proteintect, Wuhan, China, SA00001-2) were all purchased from Proteintect (Wuhan, China)

### 2.13 CCK8

Cells in the logarithmic growth phase were seeded into 96-well plates. After transfection of siRNAs, the cells were then CCK-8 reagent (Topscience, Shanghai, China) incubated and 450 nm absorbance tested at the time of 24, 48, and 72 hours after trusfection according to the manufacturer’s instructions.

### 2.14 Clone formation

Cells pre-transfected were counted and seeded into 12-well plates. After two weeks of culture, the cells were fixed with 4% paraformaldehyde, crystal violet stained, and photographed.

### 2.15 Scratch wound healing assay

Cells cultured in 6-well plates with a 60% confluency was NCAPD3 siRNAs or NC RNA transfected, scraped using a 100 μL sterile pipette tip, and gphotographed under a Nikon Ti-S fluorescence microscope at the time of 0, 12, 24, and 36 h after scrape (the same scratch area).

### 2.16 Statistical analysis

Statistical data analyses were performed using the SPSS 22.0 software. The ggplot2 package in the R programming language was used for the bioinformatic analyzing. The GraphPad Prism 9.0 software was employed to process the experimental data. The two-tailed Student’s t-test was performed to evaluate the difference between the two groups, P < 0.05 was considered to be statistically significant.

## 3 Results

### 3.1 Single-cell sequencing analysis depicts mitochondrial DNA methylation compartmentalization and breast cancer cell mapping

**Figure 1:**
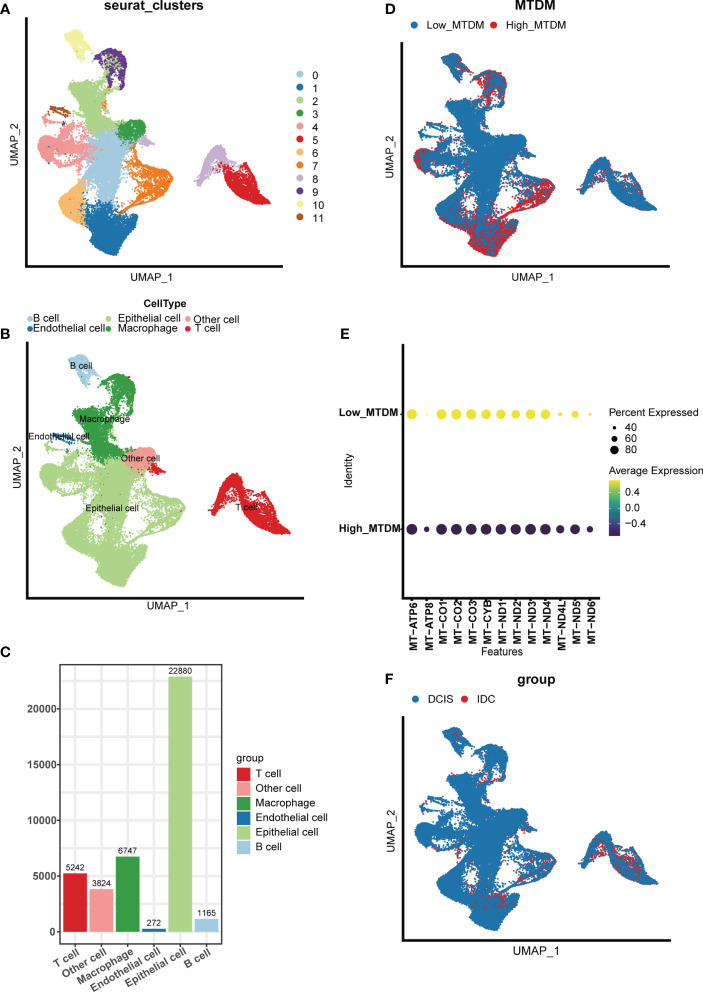
Single Cell Sequencing Data Analysis. **(A)** Dimensionality reduction and cluster analysis. All cells in 12 samples were clustered into 12 clusters. **(B)** According to the surface marker genes of different cell types, the cells are annotated as B cells, T cells, endothelial cells, epithelial cells, macrophages and other cells respectively. **(C)** Different cell types count. **(D)** The percentage of Mitochondrial DNA methylation genes in each cell. The cells were divided into high- and low-Mitochondrial DNA methylation cells. **(E)** Expression of mitochondrial coding peptides in high and low mitochondrial DNA methylation groups. **(F)** According to different cell types, breast cancer cells can be divided into Ductal carcinoma *in situ* and Invasive Ductal Carcinoma.

To investigate the modifications in mitochondrial DNA methylation (MTDM) in breast cancer (BC) cells, BC cell mapping was performed using single-cell data. After extracting and analyzing the GSE195861 dataset, 12 single-cell BC samples that were well integrated (**Supplementary Figure 1A**) and had no significant batch effect on subsequent analyses were selected. Using of “KNN” algorithm we divided the selected cells into 12 clusters (**Figure 1A**) and then annotated them according to the cell specific markers expression level (**Supplementary Figure 1B**). The annotation results showed that five cell types existed in the clusters: B cells, T cells, endothelial cells, epithelial cells, macrophages, and other cell types (**Figures 1B, C**), among which epithelial cells were mostly present, while endothelial cells were smallest (**Figure 1C**). To determine the MTDM levels in the screened cells, the annotated cells were divided into high and low MTDM groups based on the median MTDM-related gene expression (**Figure 1D**). As shown in **Figures 1B, D**, the high MTDM group was mainly concentrated in epithelial cells and macrophages. Thirteen mitochondria-encoded polypeptides are suppressed by mtDNA hypermethylation. We verified the accuracy of MTDM group division by detecting the MTDM-related genes expression. The results showed that all mitochondria-encoded polypeptides were significantly upregulated in the low-MTDM group (**Figure 1E**), consistent with the previous report. Thus, MTDM group division was reasonable. To determine the correlation between MTDM and BC malignancy, we differentiated annotated cells into ductal carcinoma *in situ* (DCIS) and invasive ductal carcinoma (IDC) cells (**Figure 1F**, **Supplementary Figure 1C**). The results showed that the epithelial cells of the more aggressive IDC group had a higher overlap with those of the high MTDM group than those of the DCIS group. Therefore, it is possible that high MTDM status contributes to BC progression.

### 3.2 BC cells with high mitochondrial DNA methylation are prone to be malignant

**Figure 2:**
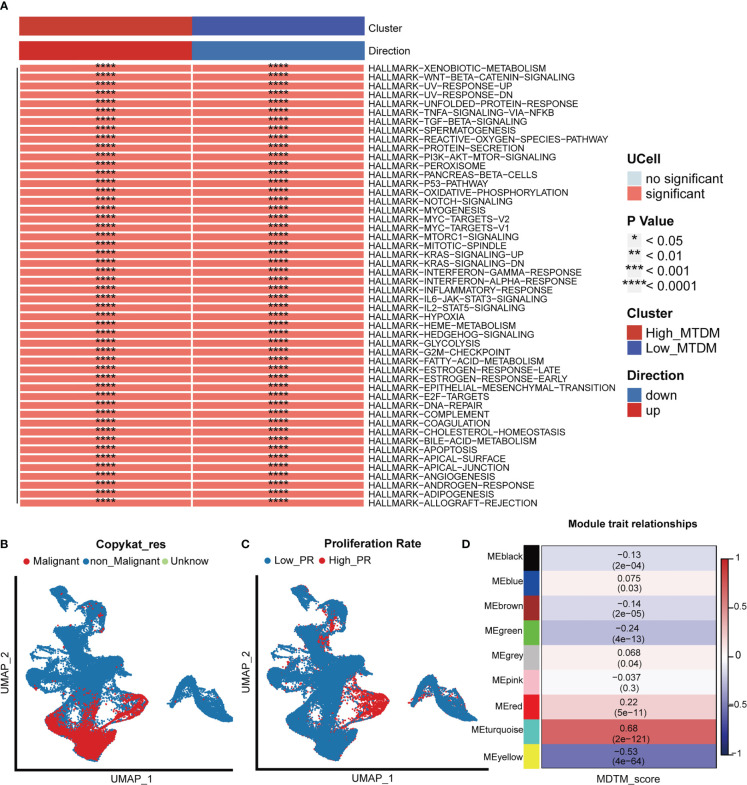
Biological differences in mitochondrial DNA methylation. **(A)** Differential biological pathway analysis showed that high and low mitochondrial DNA methylation groups were enriched in different signaling pathways. **(B)** Malignant cell identification, by chromosome integrity recognition, distinguish benign cells and malignant cells. **(C)** Cell proliferation rate was quantified by proliferation-related genes. **(D)** WGCNA found that MEturquoise, and MEyellow modules were closely related to the score of Mitochondrial DNA methylation.

As 3.1 indicated that high MTDM status contributes to the malignant progression of BC, to further explore the malignant propensity of BC cells with a high MTDM status, biological pathway enrichment analysis (**Figure 2A**) and copynumber karyotyping analysis (**Figure 2B**) were conducted. The results showed that MTDM group gene alterations occurred not only in the proliferation-related E2F signaling pathway, MYC signaling pathway, G2M checkpoint, metastasis-related WNT signaling, and epithelial mesenchymal transformation-related biological pathways, but also in apoptosis, angiogenesis, inflammatory response, and metabolism-related pathways. Additionally, copynumber karyotyping analysis (**Figure 2B**) indicated that malignant BC cells were mainly concentrated in epithelial cells, and their distribution areas highly overlapped with high MTDM areas. Considering that patients with a higher rate of tumor proliferation usually have a poorer prognosis (35), relationship between MTDM status and proliferation was further examined by distinguishing single-cells using proliferation-related markers (**Figure 2C**). The results showed that the highly proliferative regions overlapped with the high MTDM regions, indicating a malignant tendency in high MTDM status. To explore the key genes affecting MTDM, gene modules associated with MTDM were analyzed using WGCNA (**Supplementary Figures 2A–C**), and seven non-gray modules were obtained. Among these seven non-grey modules, the turquoise and yellow modules had correlations of 0.68 and -0.53 with MDTM-score, respectively, and the genes contained in these two modules were closely related to MDTM-score (**Figure 2D**). Genes with a P-value <0.001 were selected for further analysis.

### 3.3 Status of mitochondrial DNA methylation is valuable for BC prognosis

**Figure 3:**
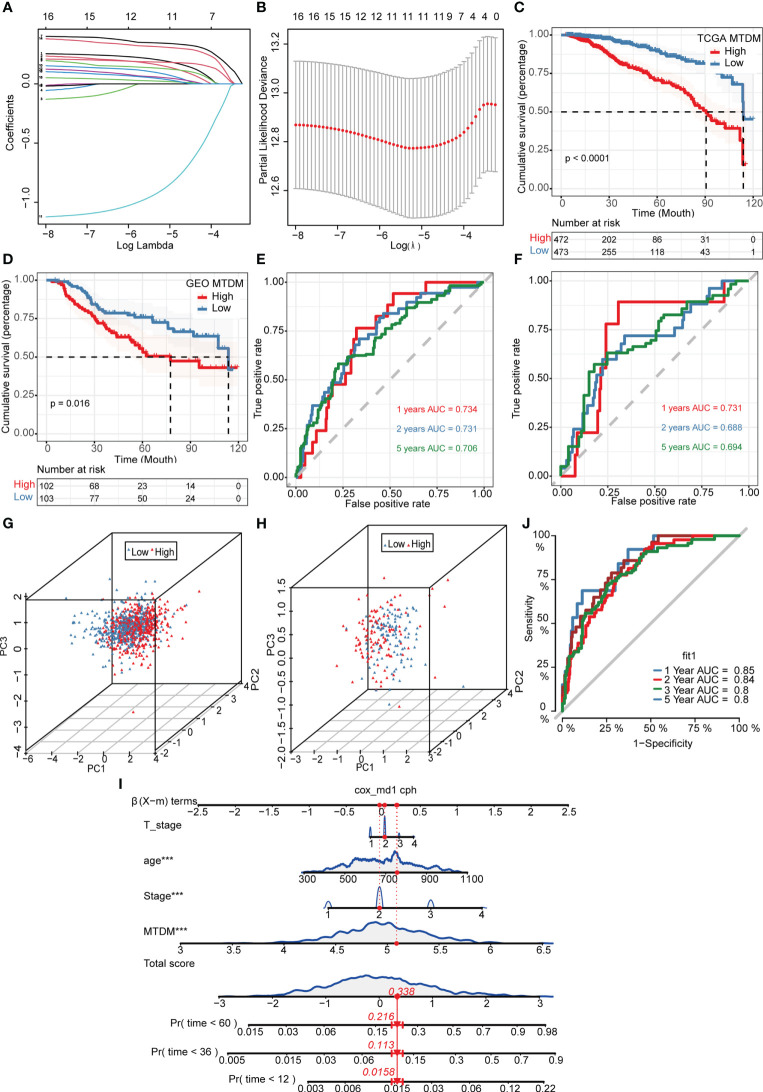
Construction and validation of Mitochondrial DNA methylation-related prognostic model. **(A, B)** sixteen genes were selected to construct the prognostic model by Lasso regression. **(C)** Survival analysis of TCGA cohort. The prognosis was significantly worse in the high-MTDM group (P<0.0001). **(D)** Survival analysis of GSE21653 Cohort. The prognosis was significantly worse in the high-MTDM group (P<0.016). **(E)** ROC curve of TCGA cohort. The AUC values of the model in 1, 2 and 5 years were 0.734, 0.731 and 0.706, respectively. **(F)** ROC curve of GSE21653 Cohort. The AUC values of the model in 1, 2 and 5 years were 0.731, 0.688 and 0.694, respectively. **(G, H)** PCA analysis of TCGA and GSE21653 queues. It was found that the model could well distinguish mitochondrial DNA methylation levels in both the training cohort and the validation cohort. **(I)** Nomogram of patient “TCGA-A2-A0CY”. The mortality rate of the patient in 1, 3 and 5 years was estimated to be 0.0158, 0.113 and 0.216. **(J)** ROC curve of the nomogram. The area under the curve (AUC) in 1,2, 3 and 5 years were 0.85, 0.84, 0.8 and 0.8 respectively. ***P<0.001.

Given the effect of changes in MTDM levels on a diverse range of biological processes, we investigated whether the genes associated with altered MTDM levels could serve as prognostic indicators in patients with BC. Thus, a total of 564 genes from the differential expression analysis of the high and low MTDM groups and WGCNA analysis of MTDM-associated genes were performed. Using univariate Cox analysis with a threshold of P < 0.05, 16 genes associated with patient prognosis were identified in the TCGA cohort. LASSO regression analysis stabilized gene contraction, and 11 genes were identified (**Figures 3A, B** and **Supplementary Table 1**). The LASSO regression results for these 11 genes are presented in **Supplementary Table 1**. Finally, we obtained a set of 11 genes, that are *ARID1B*, *B3GNT2*, *MPHOSPH10*, *NCAPD3*, *RABGAP1*, *RBM41*, *RBMXL1*, *SLBP*, *TMEM167A*, *TMEM67*, and *TUBGCP5.* To classify patients into high- and low-risk groups (i.e., high and low-MTDM groups), a prognostic model was constructed using the 11 genes and the median. Our findings revealed that the high MTDM group in the TCGA training cohort had a poorer prognosis than the low MTDM group (P < 0.0001, **Figure 3C**). The same trend was observed in the GSE21653 validation cohort (P = 0.016, **Figure 3D**). ROC curve analysis was performed in both the training and validation cohorts further evaluate the accuracy of MTDM in predicting the prognosis of patients with BC. The area under the curve (AUC) values were 0.734, 0.731, and 0.706 at 1, 2, and 5 years, respectively (**Figure 3E**) in the TCGA cohort, and 0.732, 0.686, and 0.673 at 1, 2, and 5 years, respectively (**Figure 3F**), in the validation cohort. These results indicate that the MTDM status could be used accurately to predict BC patient prognosis in both cohorts. Additionally, we conducted a PCA of the 11 genes in both the training and validation set models, and the results showed that our constructed model could discriminate between high and low MTDM in both cohorts (**Figures 3G, H**). Hereafter, high and low MTDM distinguished by risk score was used to refer the high-risk score and low risk, respectively. To better assess the risk of BC patients, a nomogram combined clinical data and MTDM values was first constructed, based on the age, T-stage, total stage and MTDM score of patients “TCGA-A2-A0CY”, our nomogram estimated a 0.0158, 0.113, and 0.216 mortality rate of patients at the year of 1, 3, and 5 years respectively (**Figure 3I**). ROC analysis was used to evaluate the accuracy of the nomogram, and the results showed that the AUC at 1, 2, 3, and 5 years were 0.85, 0.84, 0.8, and 0.8, respectively (**Figure 3J**). Thus, the constructed nomogram could effectively predict the patients with BC, and could be used to direct clinical decision-making.

### 3.4 Screen of mitochondrial DNA methylation-related prognostic genes that positively regulate breast cancer cells proliferation

**Figure 4:**
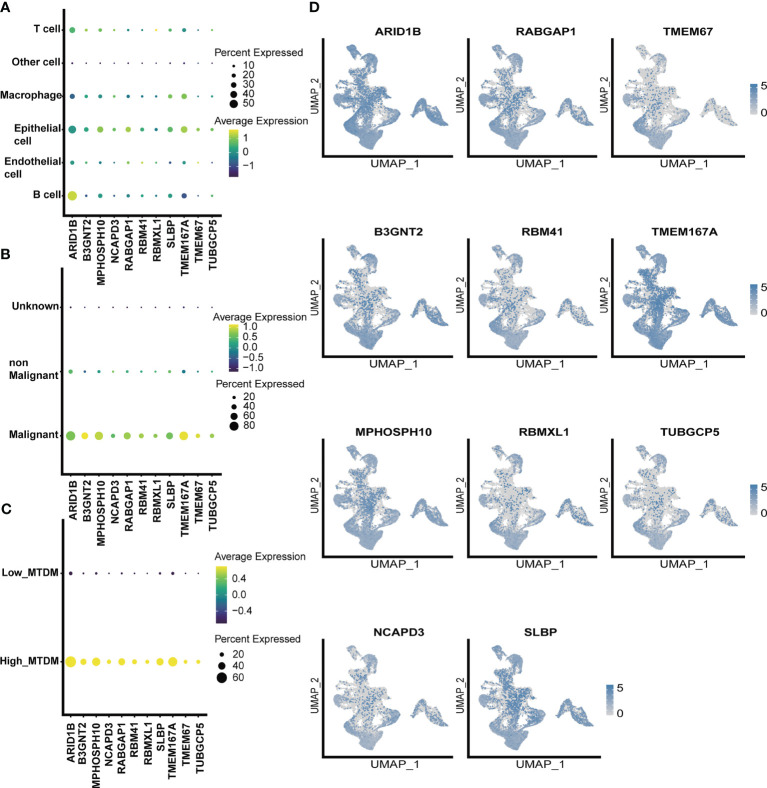
single-celled sequencing analysis, to explore the distribution of 11 modeling gene. **(A)** The expression levels of 11 modeling genes in different cell types. **(B)** The expression levels of 11 modeling genes in benign and malignant cells. **(C)** Expression levels of 11 modeling genes in high and low MTDM groups. **(D)** umap shows the expression distribution of model genes in the data set.

As altered MTDM levels may affect BC cell proliferation, further screening of MTDM-related prognostic genes that positively regulate BC proliferation could potentially be used as therapeutic targets is required. Thus, we first explored the expression of MTDM-related prognostic genes in different cell types. The results showed that all MTDM-related genes were highly expressed in epithelial cells, except for *ARID1B* which was highly expressed in B cells (**Figure 4A**). Additionally, all MTDM-related prognostic genes were highly expressed in malignant cells and cells with a high MTDM status (**Figures 4B–D**). These results are consistent with our previous results described in Section 3.3, indicating that MTDM-associated prognostic gene expression correlates well with MTDM levels in cells.

**Figure 5:**
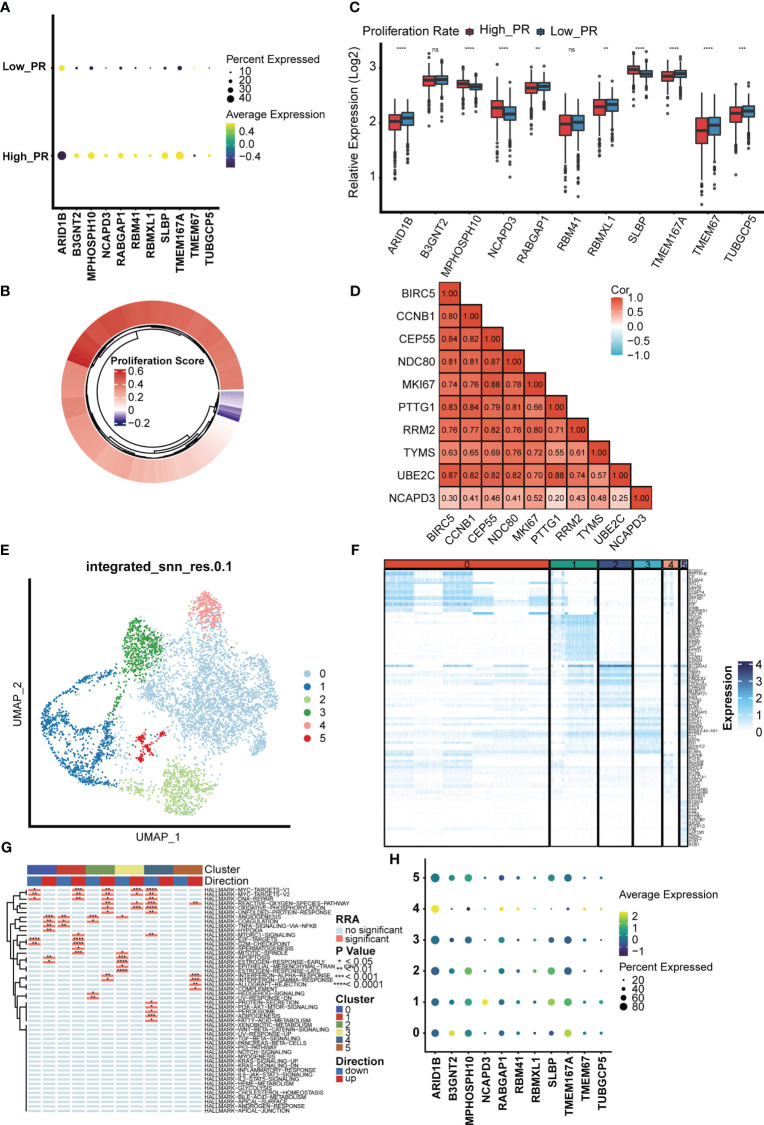
The relationship between the model gene and proliferation was analyzed in malignant cells. **(A)** Expression levels of 11 modeling genes in high and low proliferation rate groups. **(B)** The TCGA breast cancer data set was divided into high and low proliferation rate groups based on proliferation-related genes. **(C)** Expression levels of modeling genes in the high and low proliferation rate groups of the TCGA breast cancer data set. Results In the TCGA breast cancer data set, the difference in NCAPD3 was most significant between the high and low proliferation groups. ns, no significance; *p<0.05; **P<0.01; ***P<0.001. **(D)** Analysis of the correlation between NCAPD3 and proliferation-related genes showed that there was a strong correlation between NCAPD3 and proliferation-related genes. **(E)** Epithelial malignant cells were divided into 6 clusters. **(F)** Differential genes in 6 clusters showed specific expression of proliferation-related genes in cluster 1, which indicated that cluster 1 subgroup was proliferation-related epithelial malignant cells. **(G)** Differential biological pathway enrichment analysis shows the pathways enriched by different clusters. **(H)** The expression of 11 modeling genes in 6 epithelial malignant cell clusters showed that NCAPD3 had the highest expression level in cluster 1. *p<0.05; **P<0.01; ***P<0.001, ****P<0.0001.

Furthermore, we investigated the expression of MTDM prognosis-related genes in the high and low proliferation rate groups (**Figure 5A**) and found that all MTDM-related prognostic genes were highly expressed in the high proliferation rate group, except for ARID1B which was highly expressed in the low proliferation rate group. Again, we generated scores for proliferation-related markers using ssGSEA in the TCGA BC patient cohort (**Figure 5B**). The high and low proliferation rate groups were distinguished by the median proliferation-related marker scores (**Figure 5C**). The results showed that MPHOSH10, NCAPD3 and SLBP were highly expressed at high proliferation rates, with the most significant difference observed in NCAPD3. The correlation heat map results also showed that NCAPD3 had a significant positive correlation with proliferation-related markers and correlated with MKI67 at 0.52 (**Figure 5D**). We further subdivided malignant cells to focus on the relationship between MTDM-related prognostic genes and malignant cells. We identified a total of six malignant cell clusters (**Figure 5E**) and compared the signature genes of each cluster and found that cluster 1 was highly expressed in proliferation-related markers, such as MKI67, CCNB1 and UBE2C (**Figure 5F**), and GSVA analysis also showed that cluster 1 was highly enriched in proliferation-related pathways, such as the MYC signaling pathway, DNA repair pathway, E2F signaling pathway, and G2M checkpoint signaling (**Figure 5G**). This suggests that Cluster 1 is a malignant cell in a proliferative state. Finally, we investigated the expression of MTDM-related prognostic genes in a subpopulation of malignant cells and consistent with the TCGA BC patient cohort, NCAPD3 was expressed at the highest level in malignant cells in the proliferative state (**Figure 5H**). These results also demonstrate that high MTDM status may promote malignant cell proliferation, and NCAPD3 in particular, may play a key role in this process.

### 3.5 The changes of immune microenvironment suggest that the mitochondrial DNA methylation status may respond to the sensitivity of breast cancer immunotherapy

**Figure 6:**
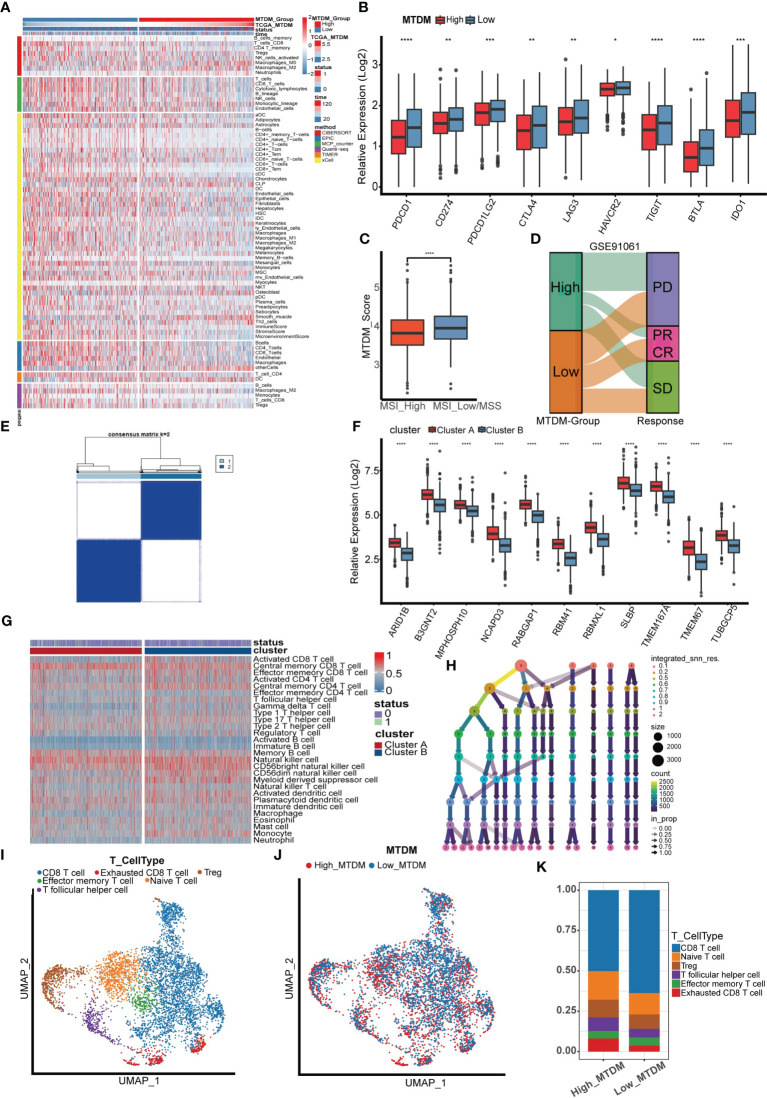
Immune infiltration analysis and immunotherapy sensitivity prediction. **(A)** Heat map of immune cell infiltration in high MTDM group and low MTDM group. **(B)** Expression of immune checkpoint related genes in high -MTDM group and low -MTDM group. **(C)** The results of microsatellite instability state analysis showed that the high MSI group had lower MTDM scores. *P < 0.05; **P < 0.01; ***P < 0.001; ****P < 0.0001. **(D)** The relationship between mitochondrial DNA methylation score and response to immunotherapy was evaluated using immunotherapy cohort GSE91061. **(E)** Unsupervised consistency cluster analysis. Patients can be divided into two clusters according to the expression of model genes. **(F)** The expression levels of model genes in different clusters. **(G)** Immune landscape of different clusters. **(H)** Decision tree analysis is used to determine the optimal clustering threshold. **(I)** According to the surface marker genes of different cell types, the cells are annotated as CD8 T cells, Exhausted CD8 T cells, T follicular helper cells, Treg cells, Effector memory T cells and Naive T cells respectively. **(J)** The percentage of Mitochondrial DNA methylation genes in T cells. The cells were divided into high- and low-MTDM cells. **(K)** Proportion of T cell types in high and low MTDM groups.

Because a high MTDM state can affect the expression of mitochondria-encoded polypeptides, preventing them from effectively maintaining the integrity of the oxidative respiratory chain (12–14), this would cause alterations in the metabolic state of tumor cells. In turn, the metabolic reprogramming of tumor cells is also aimed at promoting the rapid proliferation of tumor cells by adjusting energy metabolism (36). Since high MTDM state would inhibit OXPHOS and promote malignant cell proliferation, we hypothesized that a high MTDM state could potentially drive tumor cells to shift from OXPHOS to aerobic glycolysis, and that the low PH microenvironment created by the metabolites of aerobic glycolysis is conducive to tumor immune escape and promotes tumor progression (37, 38). Therefore, we aimed to investigate changes in the tumor immune microenvironment under different states of MTDM and whether MTDM-related prognostic genes could respond to the sensitivity of BC immunotherapy. First, we explored the differences in immune cell infiltration levels between the high and low MTDM groups. The results showed increased immune cell infiltration, including B cells, NK cells, and T cells in the low-MTDM group (**Figure 6A**). The expression of immune checkpoint-related genes was analyzed, and the results showed that the immune checkpoint-related genes included in the study were highly expressed in the low-MTDM group (**Figure 6B**). This may have resulted in low immune cell responsiveness in the low-MTDM group owing to the high expression of immune checkpoint genes despite the high level of immune infiltration. To explore whether immunotherapy is more applicable to the low-MTDM group, we calculated the MSI and TMB scores to review their relationship with MTDM. Tumors with high MSI are usually sensitive to immune checkpoint blockade (39); therefore, we chose this score. Based on the results showing that the high MSI group had a lower MTDM score (**Figure 6C**), this evidence suggests that patients in the low-MTDM group may benefit more from immune checkpoint inhibitors. However, predicting TMB sensitivity to immune checkpoint inhibitors in BC is controversial (40–43). In our results, we found a higher TMB in the high MTDM group (**Supplementary Figures 3A–C**) and a negative correlation with the degree of immune infiltration, which is inconsistent with the positive correlation between TMB and immune infiltration in most studies. Therefore, we concluded that the TMB score is not suitable as a biomarker for BC immunotherapy. To further test our prediction, we used the cohort of immunotherapy-treated melanoma patients GSE91061 (44) (**Figure 6D**). As expected, the low-MTDM group had the largest proportion of patients who achieved a CR or PR. Meanwhile, unsupervised clustering showed that MTDM related prognostic genes were best classified into two groups in the TCGA dataset (**Figures 6E, F**). There was also a significant difference in the immune cells between the two groups (**Figure 6G**), with cluster B showing lower expression of MTDM related prognostic genes and higher levels of immune cell infiltration. Finally, we differentiated T cells among the single-cell data set, and the best binning was 12 according to the decision tree (**Figure 6H**, **Supplementary Figure 3D**). T cells were classified into CD8-positive T cells, Exhausted CD8 T cells, T follicular helper cells, effector memory T cells, naïve T cells, and Tregs according to the available markers (**Figure 6I**, **Supplementary Figure 3E**). The ratio of cells between the two groups showed that the high MTDM group had a higher proportion of Exhausted CD8 T cells, a marker of immune dysfunction (45), while the low MTDM group had a higher proportion of CD8-positive T cells (**Figures 6J, K**). These results suggested that the low-MTDM group may be more responsive to immunotherapy.

### 3.6 Cellular communication reveals a potential pathway for mitochondrial DNA methylation to mediate immunosuppression

**Figure 7:**
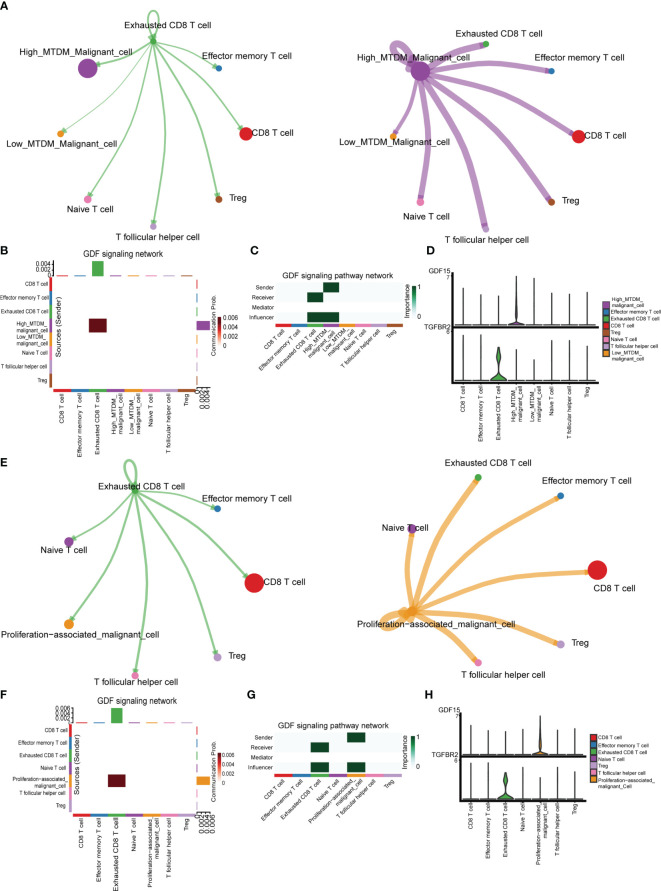
Cell communication analysis shows the interactions between different kinds of cells. **(A)** Overall communication condition of Exhausted CD8 T cells and High-MTDM Malignant cells. Circle sizes are proportional to the number of cells in each cell group and edge width represents the communication probability. **(B)** Overall activation of the GDF signaling pathway was observed in T cell subtypes and malignant cells with different MTDM levels. **(C)** The malignant cells in the high MTDM group were the only transmitters of GDF signal, and the Exhausted CD8 T cells were the only receivers of this signal. **(D)** Expression of GDF and its ligand, TGFBR2, in T cell subtypes and malignant cells with different MTDM levels. **(E)** Overall communication condition of Exhausted CD8 T cells and Proliferation-associated malignant cells. **(F)** Overall activation of the GDF signaling pathway was observed in T cell subtypes and Proliferation-associated malignant cells. **(G)** The Proliferation-associated malignant cells were the only transmitters of GDF signal, and the Exhausted CD8 T cells were the only receivers of this signal. **(H)** Expression of GDF and its ligand, TGFBR2, in T cell subtypes and Proliferation-associated malignant cells.

We explored the cellular communication characteristics between malignant cells and T cell subpopulations in different MTDM states, and the intercellular communication characteristics can effectively identify the receptor-ligand relationships that exist between cell populations, and we used this approach to uncover possible ligand-receptor pairs between high MTDM/proliferation-associated malignant cells and immune cell subpopulations as a response to the linkage between the two subpopulations and the potential mechanisms of action. **Figure 7A** shows the overall communication conditions between Exhausted CD8 T cells and high MTDM malignant cells. It was found that high MTDM malignant cells emit a *GDF15* signal. In contrast, Exhausted CD8 T cells relied on *TGFBR2*, the receptor of *GDF15*, to receive this signal (**Figures 7B, C**). In addition, the secretion of *GDF15* by high MTDM malignant cells and the expression of *TGFBR2* by Exhausted CD8 T cells were cell-specific (**Figure 7D**). *GDF15*, a member of the transforming growth factor β (TGF-β) family, inhibits the expression of co-stimulatory and major histocompatibility complex (MHC) class II molecules, it decreases IL-12 levels and increases TGF-β1 secretion (46). In hepatocellular carcinoma, *GDF15* acts as a critical promoter of Treg cells to promote Treg cell production thereby mediating immunosuppressive responses (47). Our results suggest that malignant cells with high MTDM status may cause exhaustion of CD8 T cells through the secretion of *GDF15*, leading to immunosuppression. Similarly, we explored the intercellular communication between proliferation-associated malignant cells (cluster1) and T cells. **Figure 7E** shows the overall communication between Exhausted CD8 T cells and high MTDM malignant cells. The same results showed that proliferation-associated malignant cells specifically secrete *GDF15*, and Exhausted CD8 T cells receive this signal (**Figures 7F–H**). This suggests that *GDF15* may be a key factor in the immunosuppressive response of malignant cells with a high MTDM status and proliferation rate.

### 3.7 *In vitro* experiments confirm that the MTDM-associated prognostic gene NCAPD3 does promote the proliferation of breast cancer cells

**Figure 8:**
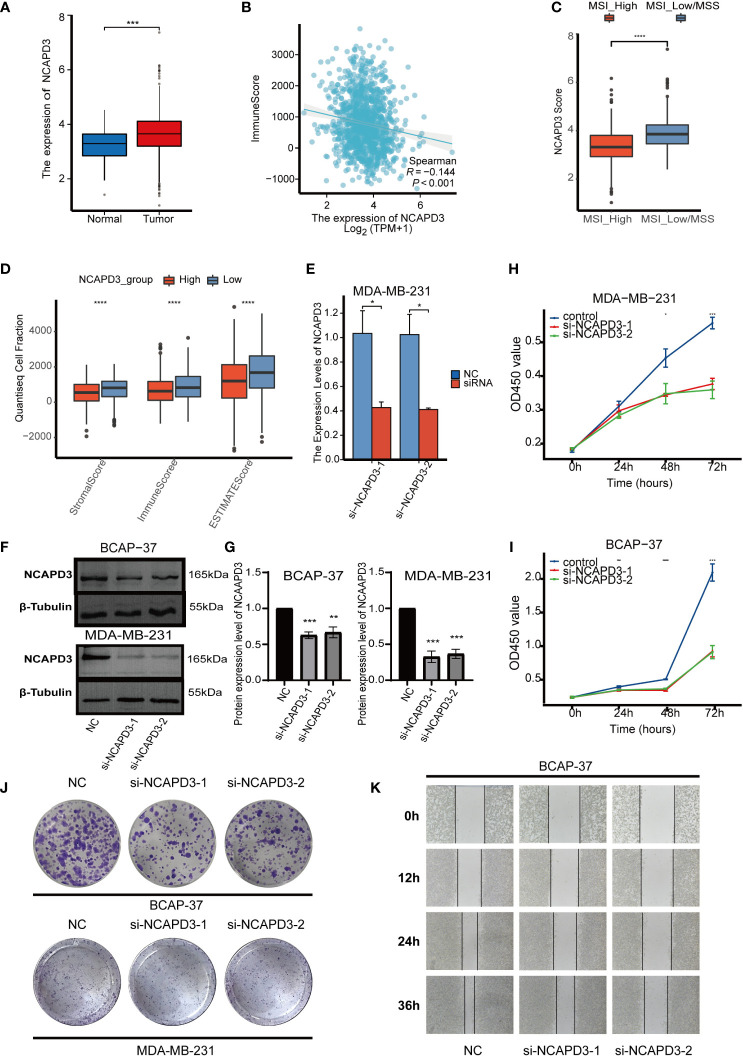
*In vitro* biofunctional validation of the MTDM-associated prognostic molecule NCAPD3. **(A)** Expression of NCAPD3 in cancer and peritumoral samples in the TCGA breast cancer dataset. **(B)** Analysis of correlation between NCAPD3 and immune score. **(C)** The results of microsatellite instability analysis showed that the high MSI group had lower NCAPD3 expression. **(D)** The estimate analysis showed the relationship between the expression level of NCAPD3 and stromal score, immune score and estimate score. **(E)** qRT-PCR to evaluate the level of NCAPD3 mRNA 1 days after transfection. All siRNA sequences could result in significant decrease in NCAPD3 mRNA expression (P<0.05). **(F-G)** Western Blot to evaluate the level of NCAPD3 proteins 2 days after transfection. All siRNA sequences could result in significant decrease in NCAPD3 proteins expression (P<0.01). **(H-I)** CCK8 assay. After NCAPD3 knockdown, the cells showed significant reduction in viability. **(J)** Colony formation assay. Cells with a reduced NCAPD3 expression exhibited a significant decrease in the numbers of colonies, compared with the siRNA negative control (NC) group. **(K)** Scratch-wound healing assay. A significantly slower wound healing rate was observed in cells with a decreased expression of NCAPD3 gene. All data were presented as the means ± SD of three independent experiments. *P < 0.05, **P < 0.01, ***P < 0.001, ****P < 0.0001.

As *NCAPD3* is expressed at the highest level in proliferation-related malignant cells and is positively correlated with proliferation-related genes, we further explored the expression of *NCAPD3* in BC and its effect on the immune microenvironment. In the TCGA transcriptome, *NCAPD3* was also highly expressed in tumors (**Figure 8A**). We then evaluated the correlation between *NCAPD3* expression and the immune score and MSI separately and found that *NCAPD3* expression was negatively correlated with the immune score and MSI (**Figures 8B–D**). Next, we performed biological functional validation of NCAPD3 knockdown *in vitro* to test whether our model gene knockdown prevents the growth of breast cancer cell lines, which could indirectly respond to whether knockdown of NCAPD3 improves the prognosis of breast cancer patients. First, we verified the mRNA levels of *NCAPD3* in the MDA-MB-231 BC cell line 1 day after transfection using q-PCR (**Figure 8E**) and found that all siRNA interference resulted in a significant reduction in *NCAPD3* mRNA expression (P< 0.05). We also verified the protein levels of *NCAPD3* in MDA-MB-231 and BCAP-37 BC cell lines after 2 days of transfection by western blotting (**Figures 8F, G**) and again found that the siRNA sequences resulted in reduced protein levels of *NCAPD3* (P< 0.01). To assess the effect of *NCAPD3* on cell proliferation, we performed CCK8 and clone formation assays. CCK8 results showed that after *NCAPD3* knockdown, cell viability was significantly reduced in MDA-MB-231 and BCAP-37 BC cell lines compared to that in the siRNA negative control (NC) group (P<0.05) (**Figures 8H, I**). The results of the cloning assay showed that the number of colonies with reduced *NCAPD3* expression was significantly reduced in both the BC cell lines (**Figures 8J, K**, **Supplementary Figure 3F**). Finally, we performed scratch assays to test whether *NCAPD3* knockdown affected the migration ability of BCAP-37 BC cell lines. The results showed that scratch healing was significantly slower in the *NCAPD3* knockdown group than in the siRNA negative control (NC) group (**Figure 8L**, **Supplementary Figure 3G**), indicating that *NCAPD3* knockdown may be an effective strategy to inhibit the proliferation and migration of BC cells. These results suggest that NCAPD3 targeting may be a desirable outcome for BC treatment and may improve patient prognosis.

### 4 Discussion

In this study, we found that altered MTDM levels play a key role in BC progression and influence the prognosis and immunotherapy outcomes of BC patients. We constructed a BC cell profile by using GSE195861 single cell data and distinguished between high and low MTDM groups, while high MTDM cells were mainly present in cells derived from IDC patients, with high proliferation rates and higher malignancy, suggesting that high levels of MTDM may be associated with the malignant trend of BC. Further investigation revealed that MTDM levels also had an impact on BC progression and patient prognostic key gene expression and were influenced by these genes regulating downstream signaling pathways. The high MTDM group showed a lower level of immune infiltration, a higher proportion of Exhausted CD8 T cells and a higher proliferation rate compared to the low MTDM group, which also had a poorer prognosis. In addition, the findings revealed that cells with low MTDM levels had a higher percentage of immune cell infiltration and a higher percentage of CD8-active T cells adapted for immunotherapy. Finally, we also analyzed the cell-to-cell communication characteristics of T cell fractions and high MTDM malignancy/proliferation-associated malignancy cells. The results showed that high MTDM malignancy/proliferation-associated malignancy cells secreted GDF15 and Exhausted CD8 T cells expressed TGFBR2, the receptor for GDF15, leading to tumor cell immunosuppression. In conclusion, our study highlights the non-negligible role of MTDM in BC progression and its impact on a wide range of biological functions. Therefore, this is an area worth exploring in BC therapy.

In the article, we identified MTDM-related molecules representative of patient prognosis and immunotherapy sensitivity to construct a portfolio of markers for predicting BC patient prognosis and immunotherapy sensitivity. These include *ARID1B*, *B3GNT2*, *MPHOSPH10*, *NCAPD3*, *RABGAP1*, *RBM41*, *RBMXL1*, *SLBP*, *TMEM167A*, *TMEM67*, and *TUBGCP5*. AT-Rich Interaction Domain 1B (ARID1B) is a component of the SWI/SNF chromatin remodeling complex. In a zebrafish model, deletion of ARID1B results in reduced body length due to dysregulation of the Wnt/β-catenin signaling pathway (48), which reveals an association between ARID1B and the Wnt/β-catenin signaling pathway. In a recent study, loss of ARID1B in ARID1A-deficient tumors destabilized SWI/SNF and impaired cancer cell proliferation (49). Meanwhile, ARID1B was identified as a potential therapeutic target in ARID1A mutant neuroblastoma (50). BetaGal Beta-1,3-N-Acetylglucosaminyltransferase 2 (B3GNT2) encodes a poly-N-acetyl lactosamine synthase that targets multiple ligands and receptors to disrupt tumor-T cell interactions and reduces T cell activation (51). M-Phase Phosphoprotein 10 (MPHOSPH10) encodes a protein that is phosphorylated during mitosis and may be involved in rRNA preprocessing (52). For fast-growing cancer cells, an active translational machinery is required to meet the needs of protein production, in which robust ribosome biogenesis plays a key role (53). Recent studies have shown that UTP11 facilitates pre-rRNA processing by binding to MPHOSPH10. When this process is blocked, it triggers nuclear stress and leads to p53 activation and cancer cell growth arrest (54). RAB GTPase Activating Protein 1 (RABGAP1) is a GTPase activating protein of RAB6A that plays a key role as a master regulator in cellular compartment localization and vesicle transport (55). Many Rab proteins are involved in cancer progression and in recent studies, RABGAP1 was shown to be regulated by Tuftelin 1 (TUFT1), promoting perinuclear lysosome accumulation and intracellular vesicle transport, which in turn is involved in tumor development (56). RNA Binding Motif Protein 41 (RBM41) is predicted to be a target gene for hsa-miR-136 (57), which has been shown to possess oncogenic effects (58), and RBM41 may also be involved in this process. RNA Binding Motif protein, X-linked Like 1 (RBMXL1) is an RNA-binding protein that may be involved in pre-mRNA splicing, and it has been reported that melanomas with RBMXL1 mutations may have correspondingly extensive Studies of Alternative RNA Splicing (ARS) (59), which may provide novel antigenic epitopes (60). stem-loop binding protein (SLBP) is a protein that binds to the histone mRNA stem-loop sequence and regulates the 3’ processing of histone mRNA (61), SLBP is required for histone biosynthesis and also for rapid cell proliferation (62), as RNAi downregulation of SLBP expression inhibits DNA synthesis and thus inhibits cycle progression (63). A recent study showed that SLBP is indeed involved in breast cancer development and that inhibition of SLBP expression would be a promising therapeutic target for breast cancer (64). Transmembrane Protein 167A (TMEM167A) is a protein associated with vesicle transport and secretion that regulates the transport of newly synthesized proteins from the ER-Golgi to the cell membrane or other organelles and is also required for glioma growth in human cell xenografts and Drosophila models, and interference with TMEM167A expression impedes a variety of tumor cell proliferation (65, 66). Transmembrane Protein 67 (TMEM67) plays a role in the migration of centrioles to the apical membrane and the formation of primary cilia (67). TMEM67 was identified as a candidate therapeutic target for triple negative breast cancer (TNBC) in a recent study, which revealed TMEM67 amplification in TNBC and proliferation inhibition of TNBC cell lines by interference with TMEM67 expression (68). Gamma-Tubulin Complex Component 5 (TUBGCP5, also known as GCP5), is involved in microtubule binding activity and microtubule nucleation (69). Recent studies have reported that TUBGCP5 can be regulated by the Long non-coding RNA Hotair, which in turn promotes the proliferation, migration and invasion of gastric cancer cells, and the regulation of the Hotair/TUBGCP5 axis may be a potential therapeutic target for gastric cancer (70). Non-SMC Condensin II Complex Subunit D3 (NCAPD3) is a subunit of the cohesin II complex, a complex of mitotic chromosomal structures involved in the physical rigidity of the chromosome spindle (71). Recent studies have shown that NCAPD3 plays a key role in CRC progression by upregulating various transcription factors, such as E2F1 and c-Myc and regulating glycolytic function in multiple ways to mediate tumorigenesis and progression (72). In prostate cancer, NCAPD3 has also been shown to activate E2F1 and STAT3 and drive prostate cancer progression (73). In addition, NCAPD3 is present in the outer mitochondrial membrane and regulates oxidative stress in the mitochondria, which is not dependent on glycolysis and does not affect the number of mitochondria (74). Ultimately, *in vitro* experiments also confirmed that the knockdown of NCAPD3 resulted in a significant decrease in cell viability and colony number in BC cell lines MDA-MB-231 and BCAP-37 and a significant slowdown in scratch healing in MDA-MB-231 cells. Thus, our results suggest that NCAPD3 is a promising therapeutic target for BC and further studies are needed to explore its potential.

In BC, it is commonly observed that BC possesses lower mtDNA content compared to standard breast specimens (75, 76), and it has also been shown that the reduction of mtDNA in BC cell lines may be associated with the conversion to a mesenchymal phenotype (77). Furthermore, MTDM largely determines the mtDNA expression. The role of MTDM in BC is almost unknown; therefore, it is necessary to analyze the specific role of MTDM in BC, and further research is needed to understand the role of MTDM and its potential as a therapeutic target.

In previous studies, the exploration of MTDM focused on its presence and alteration in different diseases (17),leading to a lack of clarity on the one hand as to why this alteration occurs, and on the other, as to what impact this alteration will have on the development of the disease. Recently, through methodological and functional studies, the academic community established MTDM as an important research direction in mammalian mitochondrial physiology (78). Previous methodologies have been dedicated to the more precise identification of MTDM modification sites (22), and thus to determining the overall level of MTDM. We quantified the overall level of MTDM by collecting genes that positively regulate MTDM and quantifying the overall level of MTDM based on their expression levels, and the expression of mitochondria-encoded peptides similarly confirmed the validity of our approach. The accuracy of this method needs to be validated in combination with other MTDM sequencing methods.

In this study, we present the first systematic demonstration of the role of MTDM in breast cancer and explore its relationship with immunity and prognosis. This study provided new insights into the use of BC immunotherapy. We found that MTDM-related prognostic molecules, particularly *NCAPD3*, were strongly positively correlated with proliferation. *In vitro* knockdown of *NCAPD3* interfered with cell viability, clonogenic capacity, and migration ability of BC cell lines, suggesting that *NCAPD3* may be a promising target for BC therapy. This demonstrates that interference with the MTDM pathway has great potential for cancer therapy. In particular, the understanding of the importance of MTDM in mammalian mitochondrial physiology has strengthened research in this direction. Thus, intervention of the MTDM pathway is a novel cancer treatment strategy that will lead to better patient outcomes. Our findings provide a potential target for BC therapy; however, further studies are needed to explore this potential.

## Data availability statement

Publicly available datasets were analyzed in this study. This data can be found in TCGA database, GEO database, GSE195861, GSE21653, and GSE91061.

## Author contributions

Conception and design: JZ, YM, JD, MC, and XW. Acquisition of data: YM, MC, and NG. Data analysis and interpretation: YM, JD, and SW. Draft and manuscript: YM, JD, and ZM. All authors contributed to the article and approved the submitted version.

## Supplementary material

Supplementary Figure 1 Single cell sequencing analysis of GSE195861. **(A)** The integration effect of 12 samples is good. **(B)** Cell numbers in ductal carcinoma *in situ* and invasive ductal carcinoma samples. **(C)** Annotated markers of different kinds of cells.

Supplementary Figure 2 WGCNA analysis. **(A)** Cluster tree of TCGA breast cancer samples to exclude outliers. **(B)** Find the optimal soft threshold. **(C)** Cluster Dendrogram, used to find correlations between modules and samples.

Supplementary Figure 3 TMB analysis and T cell isotype annotation. **(A)** Mutation landscape of high-MTDM group. **(B)** Mutation landscape of low-MTDM group. **(C)** TPM scores of high and low MTDM groups. **(D)** Dimensionality reduction and cluster analysis. All T cells were clustered into 12 clusters. **(E)** Annotated markers of different kinds of T cells. **(F)** Quantitative diagram of clone formation experiments. **(G)** Experimental quantified graph of scratch healing ability.
