# Supplementary figures and images for: Mitochondrial DNA methylation is a predictor of immunotherapy response and prognosis in breast cancer: scRNA-seq and bulk-seq data insights
Source: Front Immunol. 2023 Jun 29;14:1219652. doi: 10.3389/fimmu.2023.1219652 (PMC10339346; doi:10.3389/fimmu.2023.1219652)

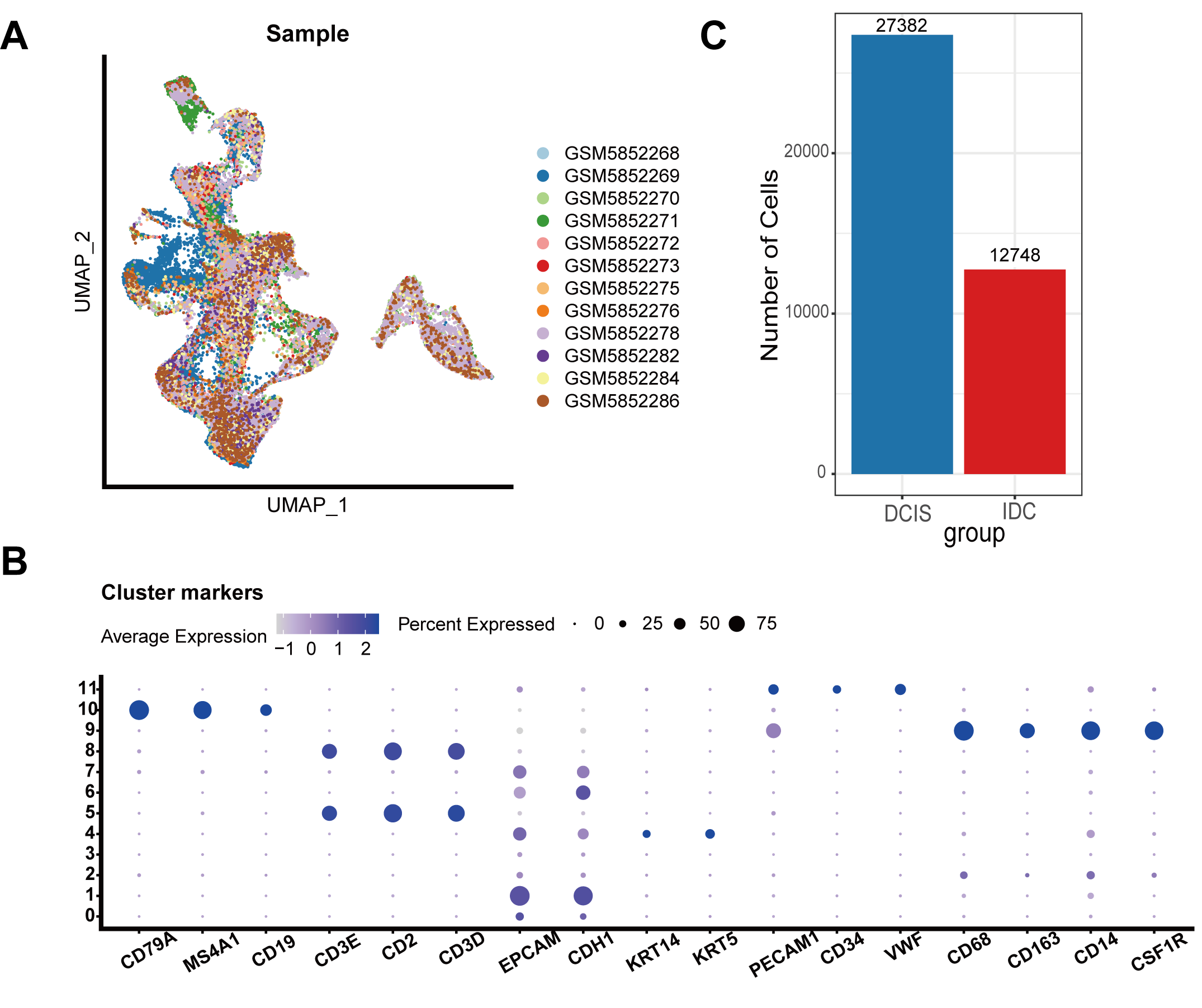

Supplement: Supplementary Figure 1 — Single cell sequencing analysis of GSE195861. (A) The integration effect of 12 samples is good. (B) Cell numbers in ductal carcinoma in situ and invasive ductal carcinoma samples. (C) Annotated markers of different kinds of cells. [file Image_1.tif]

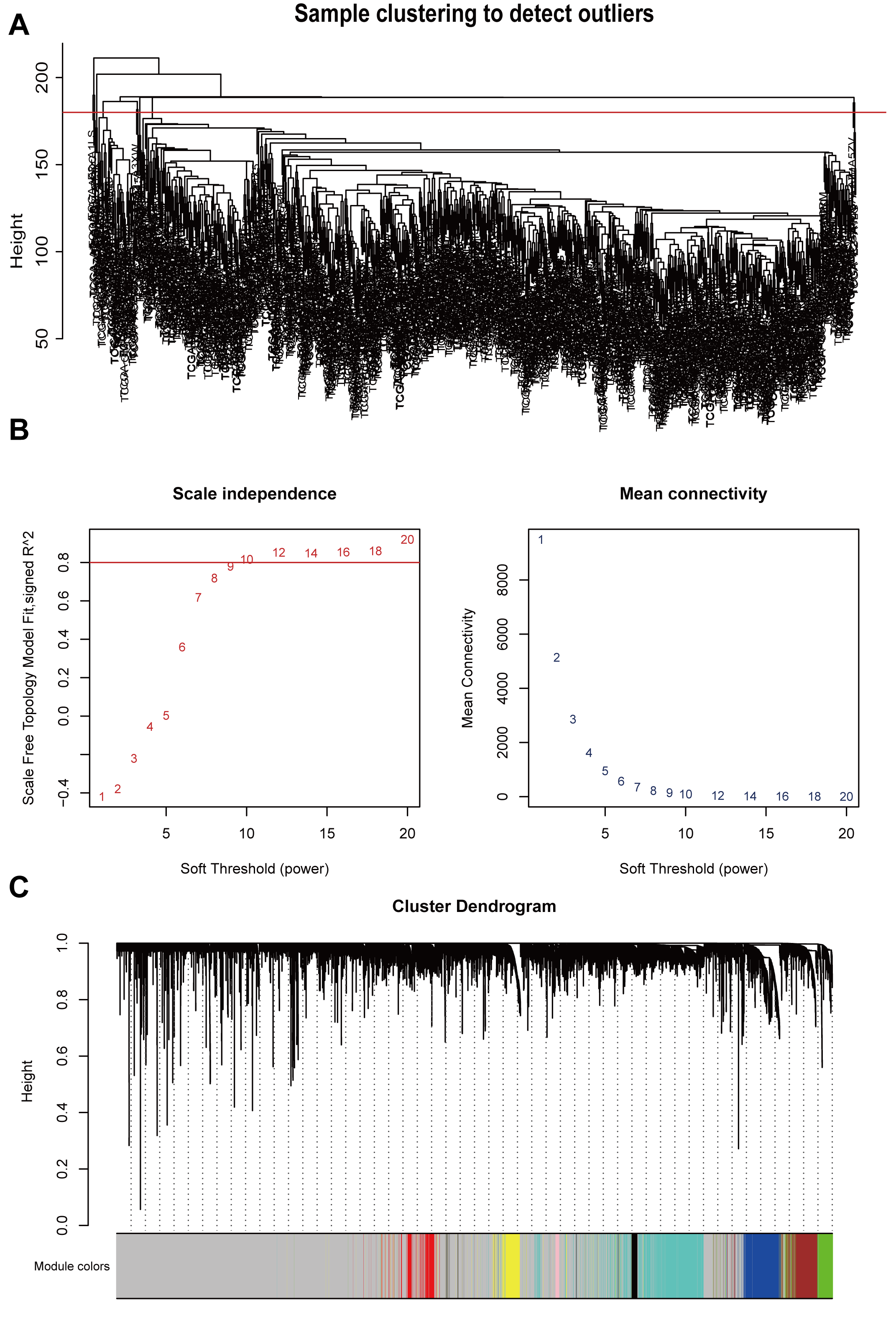

Supplement: Supplementary Figure 2 — WGCNA analysis. (A) Cluster tree of TCGA breast cancer samples to exclude outliers. (B) Find the optimal soft threshold. (C) Cluster Dendrogram, used to find correlations between modules and samples. [file Image_2.tif]

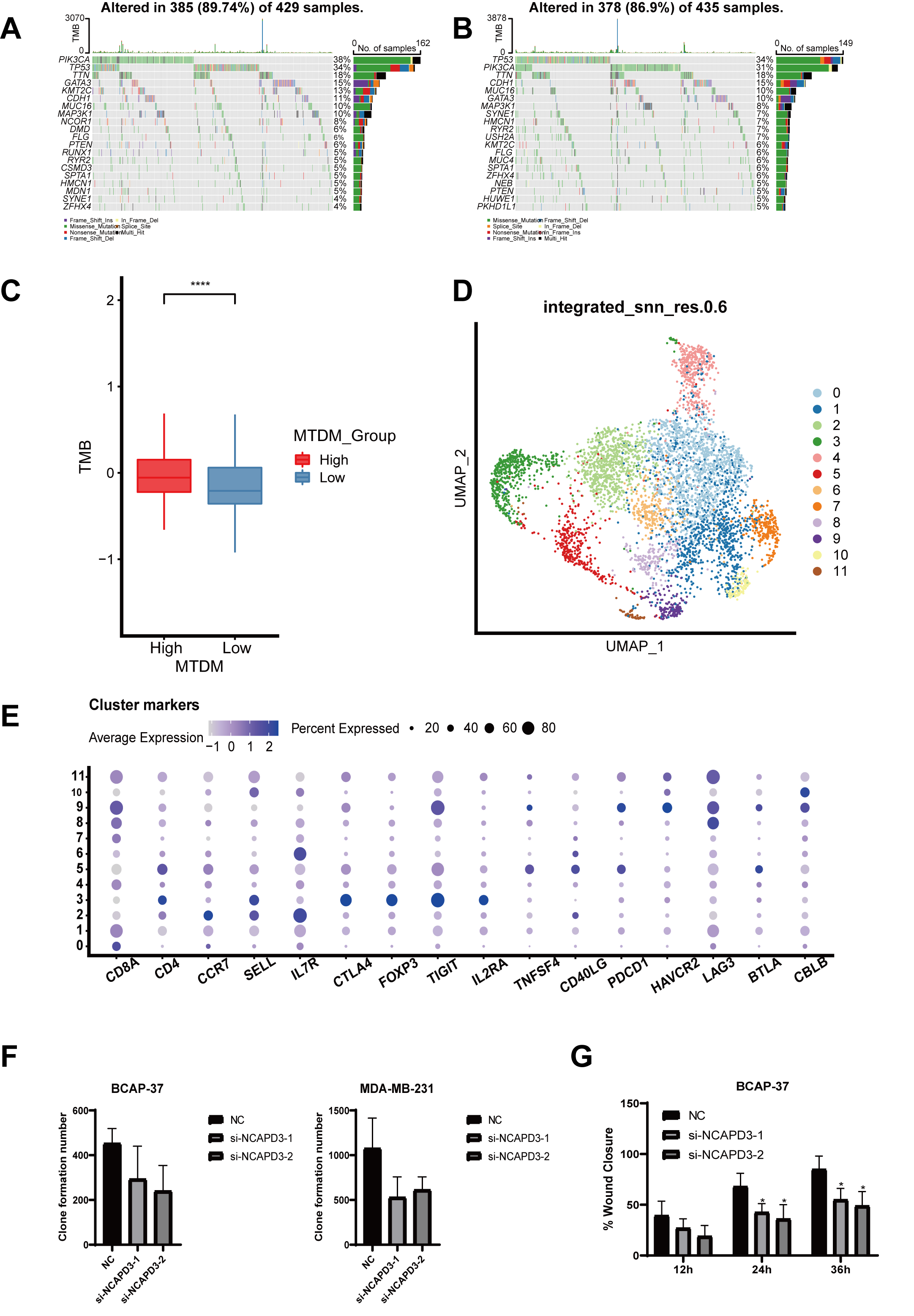

Supplement: Supplementary Figure 3 — TMB analysis and T cell isotype annotation. (A) Mutation landscape of high-MTDM group. (B) Mutation landscape of low-MTDM group. (C) TPM scores of high and low MTDM groups. (D) Dimensionality reduction and cluster analysis. All T cells were clustered into 12 clusters. (E) Annotated markers of different kinds of T cells. (F) Quantitative diagram of clone formation experiments. (G) Experimental quantified graph of scratch healing ability. [file Image_3.tif]
